# How to Detect Antibodies Against *Babesia divergens* in Human Blood Samples

**DOI:** 10.1093/ofid/ofae028

**Published:** 2024-01-16

**Authors:** Muyideen Kolapo Tijani, Joel Svensson, Paula Adlerborn, Lena Danielsson, Alexandra Teleka, Matilda Ljungqvist Lövmar, Per-Eric Lindgren, Pia Forsberg, Kristina E M Persson

**Affiliations:** Department of Laboratory Medicine, Lund University, Lund, Sweden; Department of Laboratory Medicine, Lund University, Lund, Sweden; Clinical Chemistry and Pharmacology, Laboratory Medicine, Office for Medical Services, Region Skåne, Lund, Sweden; Laboratory Medicine, Unilabs, Skaraborg Hospital Skövde, Skövde, Sweden; Department of Laboratory Medicine, Lund University, Lund, Sweden; Clinical Chemistry and Pharmacology, Laboratory Medicine, Office for Medical Services, Region Skåne, Lund, Sweden; Department of Laboratory Medicine, Lund University, Lund, Sweden; Clinical Chemistry and Pharmacology, Laboratory Medicine, Office for Medical Services, Region Skåne, Lund, Sweden; Department of Laboratory Medicine, Lund University, Lund, Sweden; Department of Clinical Microbiology, Region Jönköping County, Jönköping, Sweden; Division of Inflammation and Infection, Department of Biomedical and Clinical Sciences, Linköping University, Linköping, Sweden; Department of Clinical Microbiology, Region Jönköping County, Jönköping, Sweden; Division of Inflammation and Infection, Department of Biomedical and Clinical Sciences, Linköping University, Linköping, Sweden; Division of Infectious Diseases, Department of Biomedical and Clinical Sciences, Linköping University, Linköping, Sweden; Department of Laboratory Medicine, Lund University, Lund, Sweden; Clinical Chemistry and Pharmacology, Laboratory Medicine, Office for Medical Services, Region Skåne, Lund, Sweden

**Keywords:** antibody, *Babesia*, blood transfusion, *divergens*, ELISA

## Abstract

**Background:**

Today only indirect fluorescent antibody assays (IFAs) are commercially available to detect antibodies against *Babesia divergens* in humans. IFA is subjective and requires highly experienced staff. We have therefore developed an enzyme-linked immunosorbent assay (ELISA)–based method for measuring anti–*B. divergens* immunoglobulin G antibodies in human blood samples.

**Methods:**

Crude merozoite extract from in vitro cultures of a new *B. divergens* isolate was used in ELISA to detect antibodies in different sets of samples: *Borrelia burgdorferi*–positive samples, healthy individuals, tick-bitten individuals including follow-up samples 3 months later, positive control samples from patients with an active *Babesia* infection, and samples from malaria-endemic regions. As a reference, IFA was used to detect antibodies in the tick-bitten samples. Western blot was used to evaluate reactions against specific bands in extracts with/without parasites.

**Results:**

Using IFA as the reference method, the sensitivity and specificity of the ELISA were 86% (12/14) and 100% (52/52). There was a very high correlation (*r* = −0.84; *P* = .0004) between IFA dilution factors and ELISA absorbances among the samples classified as positive. Five percent of the *B. burgdorferi*–positive samples were judged as weakly positive and 5% as strongly positive in our ELISA. Western blot showed that the immunodominant antigens (∼120 kDa) were from merozoites and not from erythrocytes.

**Conclusions:**

This ELISA can detect antibodies directed against *B. divergens,* and it can be a useful and easy assay to handle compared with IFA. The ELISA can also measure high and low levels of antibodies, which could give insight into the recency of a *B. divergens* infection.

In Europe, *Babesia divergens* seems to be the most frequently diagnosed *Babesia* in humans, but other *Babesiae* have also been seen [1, 2]. In Sweden, several severe cases have been described [3, 4], and *Babesia* has also been detected in ticks [5]. Individuals seropositive for *Borrelia burgdorferi*, another tick-transmitted pathogen, have been shown to have a seroprevalence of 16% for *Babesia* [6]. The climate in Southern Sweden is relatively mild, and there are plenty of animal reservoirs, creating an environment where spread of the parasites is facilitated, and babesiosis is today considered an emerging disease [7, 8].

The symptoms of babesiosis are best known in North America, with >2000 cases reported every year [9]. The risk of developing severe babesiosis increases with age, reduced splenic function, and immunosuppression. Patients often present with periodic fever, chills, anemia, and sometimes dark or red urine due to hemolysis. In severe cases, organ failure and derangements of the blood coagulation system can appear, and the patient has a major risk of dying [1, 10].

The parasites can also spread via blood transfusion; in the United States >200 cases of transfusion-transmitted babesiosis have occurred [11], and the parasites can easily survive under conditions used to store blood in blood banks [12].

The methods used in clinical practice to diagnose and detect babesiosis are rather poorly developed in most countries. Unspecific routine biochemistry may indicate general signs of infection, as well as elevated liver enzymes and hemolysis [1, 13]. Blood smear is the most common method through which *Babesia* spp. are identified [14, 15]. Inexperience could cause misidentification of *Babesia* as *Plasmodium*, especially in areas of co-endemicity. Another method for direct detection of *Babesia* parasitemia is polymerase chain reaction (PCR) [16], which can detect low parasitemias. In some states in the United States, screening of every bag of donated blood is performed using PCR [11, 17] to detect *Babesia* species including *B. microti,* the dominating parasite in these areas.

Indirect fluorescent antibody assay (IFA) is the most commonly used serological method to detect antibodies produced against *Babesia* both for clinical and research purposes, and it is the only commercially available method for detecting anti-*Babesia* antibodies in humans. The downside is that it requires highly experienced staff to interpret what is seen in the microscope, and the method is subjective [18]. Earlier, enzyme-linked immunosorbent assay (ELISA) protocols were tested for serological monitoring of bovine babesiosis and for human *B. divergens*, where a broad spectrum of antigen sources was used, for example, metabolic or soluble antigens, harvested from culture supernatants [19, 20]. The invasion-inhibitory effect of immune serum obtained from animals immunized with antigens recovered from culture supernant showed that these antigens were important for red blood cell (RBC) invasion [21]. Most clinical and research-focused ELISA protocols make use of a single recombinant antigen, but a recent study demonstrated antigenic hierarchy among *B. microti* antigens such that the highest sensitivity and specificity were obtained through a combination of multiple antigens [22]. Interestingly, most of the immunodominant antigens that gave the best sensitivity had epidermal growth factor (EGF)–like domains similar to those in merozoite surface proteins (MSPs) of *Plasmodium falciparum,* and they were indeed localized on the merozoite surface of *B. microti* [22].

Despite the epidemiological importance of *B. divergens*, there are no ELISA kits available that can be used for immunoepidemiological studies of this important parasite. It has been found earlier that culture supernatants of *B. divergens* are replete with merozoites [21, 23]. Merozoites harvested from culture supernatants as antigens in ELISAs could be a relatively cheap source of broad-spectrum antigens. Another advantage of this approach is the possibility of detecting cross-reactive antibodies produced against other *Babesia* parasites, as suggested by an earlier study in bovines [24], but also in humans where antibodies against *B. divergens* and *B. venatorum* have been shown to be higly cross-reactive, while there was very low cross-reactivity between *B. divergens* and *B. microti* [25].

Thus, the intention of this study was to create a simple and reproducible ELISA for *B. divergens* that can be used both in routine clinical practice and for research purposes. We established a method to purify *Babesia* merozoite antigen extracts from in vitro cultures to avoid reactions of autoantibodies against RBC, which could occur when extract from whole infected RBC, whether from humans or from animals, is used in IFA. This extract was utilized to set up an ELISA method, which was used to test different sample sets including samples obtained in a small seroprevalence study for *B. divergens* in Sweden.

## METHODS

### Bovine *B. divergens* Isolate and In Vitro Culture in Human RBC

A new isolate of *Babesia* (Lund 1) was introduced into culture medium containing O + erythrocytes (4% hematocrit) in 25-mL flasks and cultured at 37°C in candle light boxes, as recently described [26], and similar to what has been used for *P. falciparum* cultures before [27, 28]. The culture medium contained 1% Albumax II (Gibco), 5 mM L-glutamine (Gibco), 25 μg/mL gentamicin (Sigma), and 200 μg/mL hypoxanthine (Sigma) in RPMI 1640-HEPES (Gibco).

### Identification of Species

Samples from the cultures were sent to the Public Health Agency of Sweden, which forwarded them to University of Zurich (Institute of Parasitology, University of Zurich), where PCR confirmed the parasites to be *B. divergens*.

### Merozoite Extract Preparation

*B. divergens* strain Lund 1 was used. Supernatants from cultures with 30%–40% parasitemia were centrifuged at 2400 rpm for 3 minutes then transferred into 50-mL Falcon tubes and centrifuged at 5500 rpm for 12 minutes to pellet the merozoites. The supernatant was removed until 5–10 mL was left; the pellet was resuspended, filtered through 1.2 µm Acrodisc 32 mm (Pall Corporation), centrifuged at 13 000 rpm for 7 minutes, washed in phosphate-buffered saline (PBS) × 4, and finally resuspended in 100 µL PBS. Merozoites from several rounds were pooled and freeze-thawed × 4 to create a reproducible extract, and protein concentration was determined using a NanoDrop spectrophometer (280 nM).

### RBC Extract Preparation

Four percent hematocrit O + RBC was put overnight in culture medium at 37°C in candle light boxes, then pelleted, washed × 2 PBS, resuspended in PBS, and freeze-thawed × 4. Protein concentration was determined as above.

### Study Cohorts, Serum Samples

The healthy control group (CG) was comprised of 189 healthy volunteers age 18–67 years from Sweden [6].

The *Borrelia* antibody–positive group (BAG) was comprised of 100 patients positive for antibodies against *Borrelia burgdorferi sensu lato*, age 5–87 years, the Microbiology Biobank, Lund. Samples that were considered seropositive for *B. burgdorferi s.l.* had either elevated immunoglobulin G (IgG) concentrations (≥30 AU/mL, chemiluminescent immunoassay, LIAISON *B. burgdorferi*, DiaSorin) or clearly elevated IgM indexes (>1.1 AU/mL). An immunoblot assay (EUROLINE-WB Euroimmun AG) was also performed on the samples with elevated IgM indexes for confirmation. This 2-tier protocol is in accordance with current standard recommendations [29].

There were 66 samples from 33 tick-bitten Swedish individuals (Söderhamn, STING-study), with 3 months between the first and second samples [30, 31].

As control samples, we used anonymous samples: 5 from Ugandans with acute malaria and 10/7 from healthy individuals living in malaria-endemic areas in Uganda/Nigeria, respectively. Five samples were from individuals admitted to the Infectious Disease Clinic Sweden, with different and unknown diagnoses.

We also had access to 1 plasma sample from an individual with a *B. crassa*–like infection [32] and 1 positive control sample from the Fuller IFA kit. All the samples were stored at −80°C.

The study was approved by the Regional Ethical Board, Lund, Sweden (2014/659), and the Regional Board for Quality Register (S-KVB).

### Enzyme-Linked Immunosorbent Assay for *B. divergens* Using Merozoite Extract

Maxisorp immunoplates (Thermo Fisher Scientific) were coated overnight at 4°C with 50 μL/well of merozoite extract in PBS (1.1 μg protein/well). Plates were washed × 3 with PBS/0.05% Tween 20 and then blocked with 200 μL/well of Blocker Casein (Thermo Fisher Scientific) for 2 hours (RT). Plates were washed × 3, and 50 μL of serum sample 1:50 in 0.1% Blocker Casein in wash buffer was added and incubated for 2 hours, washed × 3; 50 μL (1:10 000) of rabbit-antihuman IgG, heavy chain antibody (Thermo Fisher Scientific) in dilution buffer was added and incubated for 1 hour, washed × 3, followed by addition of 50 μL (1:3000) of goat-antirabbit IgG(H + L)–HRP (BioRad) in dilution buffer, incubated for 1 hour, washed × 3; 100 μL/well TMB One Solution (Promega) was incubated for 4 minutes and the reaction stopped with 100 μL/well of 1M H_2_SO_4_. Absorbance was read at 450 nm (Multiskan Sky, Thermo Fisher Scientific). Positive/negative controls were included in all plates, and mean values were calculated from duplicate readings.

### Indirect Fluorescent Antibody Assay

*B. divergens* IgG antibodies were detected in IFA (Fuller Laboratories, Fullerton, CA, USA) according to the manufacturer's instructions plus an extra wash step (ultra clean water), allowing wells to dry before adding conjugate/mounting medium. We used 66 samples from the STING study for IFA [30, 31].

### Western Blot

Merozoite/RBC extracts were separated on SDS-PAGE gels (Bolt 4%–12% Bis-Tris Plus Gel 1 mm, 10w, Invitrogen) using MES Running Buffer (Invitrogen). Separated proteins were transferred to polyvinylidene difluoride (PVDF) membranes (iBlot2 Dry Blotting System, Invitrogen). Blotted membranes were blocked with TBST (20 mM Tris pH 7.5, 150 mM NaCl, 0.1% Tween 20) + 5% (w/v) skim milk (Semper) o/n at 4°C, washed with TBST 3×, 5 minutes each time. Serum/plasma samples were diluted 1:50 in TBST + 3% (w/v) skim milk and incubated with the membrane for 1 hour, RT. After washing as above, rabbit-antihuman IgG, Fc (Sigma) 1:5000 in TBST + 3% (w/v) skim milk was added and incubated for 1 hour at RT. After washing, goat-antirabbit IgG (H + L)–HRP (BioRad) 1:3000 TBST + 3% (w/v) skim milk was incubated for 1 hour, RT. Following washing, bands were developed (SuperSignal West Pico Chemiluminescent Substrate, Thermo Fisher Scientific) and documented with the ChemiDoc Imaging System (BioRad). The gel after transfer and the membrane were stained with Imperial Protein Stain (Thermo Fisher Scientific) to evaluate transfer efficiency. iBright Prestained Protein Ladder (ThermoFisher) was used as a molecular size marker.

### Statistics and Quality Controls

Analyses were performed with Excel (Microsoft), GraphPad Prism 9, and SPSS, version 19.0 (SPSS Inc., Chicago, IL, USA). The Mann-Whitney test was used to compare medians between groups. Spearman's correlation was used to determine the relationship between IFA and ELISA results.

## RESULTS

### Description of Study Cohort Samples

In the healthy control group (CG), 189 individuals were included aged 18–67 years, with 66% females and 34% males, mean age 40. In the *Borrelia* antibody–positive group (BAG), 100 individuals 5–87 years of age were included with 42% females and 58% males, mean age 55.

### Long-term In Vitro Culture of *B. Divergens* and Merozoite Purification

**Figure 1.**
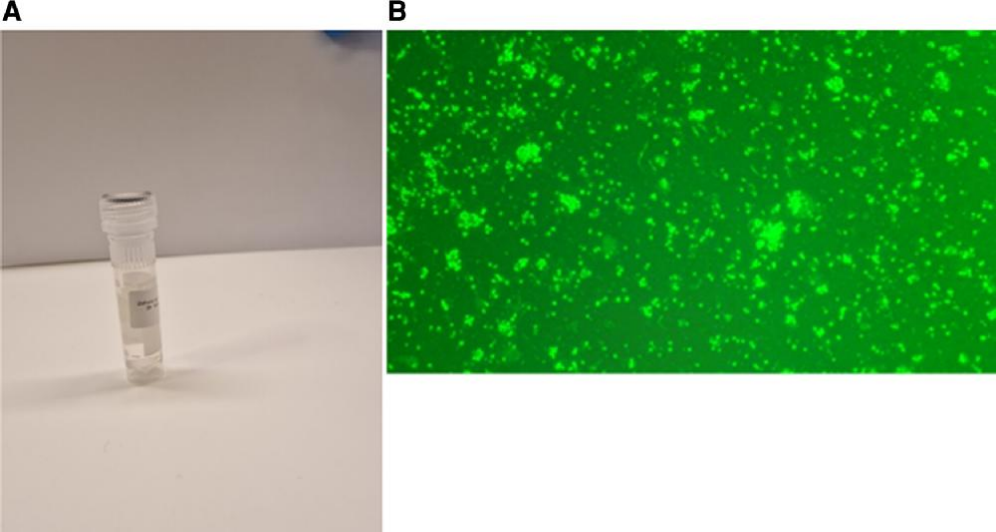
Suspension of purified *B. divergens* merozoites in PBS (A). Acridine orange-stained *B. divergens* merozoites purified from culture supernatants (B). Abbreviation: PBS, phosphate-buffered saline.

Twenty-four hours after the addition of human RBC to cow RBC, *Babesia* parasites started to invade the human RBC and could be seen as trophozoites and other stages, as shown elsewhere [26]. This *B. divergens* isolate, called Lund 1, has now been stable in continuous culture for >2 years [26]. It was used to purify merozoites according to the protocol described in the Methods section, and the results can be seen in Figure 1A. In Figure 1B, an abundance of merozoites can be seen when the purified material was stained with acridine orange.

### Anti-*Babesia* IgG Antibodies in CG and BAG

When using our ELISA, the absorbances from the samples in the control group ranged from 0.05 to 2.69 with a mean of 0.29. This group was used to calculate cutoff levels for what to consider a positive sample. An optical density (OD) value of ≥0.97 was obtained from the mean +2 SDs and was considered the limit for a weakly positive sample. A cutoff value of ≥1.3 (mean + 3 SDs) was considered strongly positive. Using the above limits, 4/189 of the CG samples (2%) were judged as being weakly positive and 4/189 (2%) as strongly positive (Table 1). Among the *Borrelia*-positive (BAG) group, the absorbances ranged from 0.09 to 2.63 with a mean of 0.38. Five of 100 (5%) were considered weakly positive and 5/100 (5%) strongly positive (Table 1).

**Table 1.** Proportion of Samples Positive for Anti–*B. divergens* Antibodies by ELISA

	No.	Weakly Positive, % (No.)	Strongly Positive, % (No.)
STING (tick-bitten)	66	-	18.2 (12/66)
BAG	100	5 (5/100)	5 (5/100)
CG	189	2 (4/189)	2 (4/189)
Infectious dis clinic	5	-	20 (1/5)
Ugandan acute malaria	5	-	-
Healthy Ugandan/Nigerian	17	-	5.9 (1/17)
*B. crassa*–like sample	1	-	100 (1/1)
Fuller kit pos control	1	-	100 (1/1)

Abbreviations: BAG, *Borrelia*-positive; CG, Swedish control samples; ELISA, enzyme-linked immunosorbent assay.

There was no detectable association between antibodies produced against *B. divergens* and age (*P* = .50), and there was no difference in median antibody levels between males and females (*P* = .92).

### Patient Samples

One sample from a patient with an active infection with a crassa-like *Babesia* was clearly positive in our ELISA, with an absorbance of 3.98.

The Fuller positive control showed a very high absorbance in our ELISA of 4.0.

### Correlation Between IFA and ELISA

**Figure 2.**
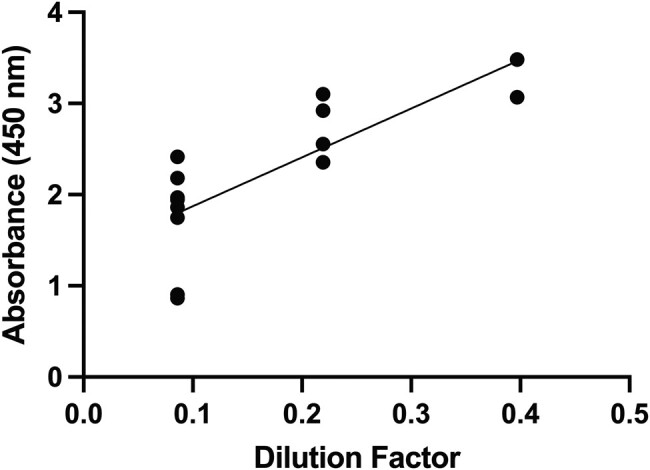
Spearman's correlation between IFA dilution factors and absorbance (antibody levels) obtained from ELISA (*r* = 0.84; *P* = .0004). Abbreviations: ELISA, enzyme-linked immunosorbent assay; IFA, indirect fluorescent antibody assay.

When using IFA for the STING study samples, 14 samples (from 7 individuals) were considered positive (Figure 2). There was a very high correlation between IFA dilution factors and ELISA results when using the positive STING study samples (*r* = 0.84; *P* = .0004) (Figure 2). Using 1.3 as cutoff to assess these samples, our ELISA protocol showed a sensitivity of 86% (12/14) and a specificity of 100% (52/52).

### ELISA Results in Different Groups

When using samples from malaria-endemic areas (Uganda and Nigeria), all acute malaria samples were negative, while only 5.9% (1/17) of samples from healthy individuals were positive. For the 5 samples obtained from the Infectious Disease Clinic in Sweden, 1 sample was judged as being positive (Table 1). Supplementary Figure 1 shows the distribution of antibody levels in the different sample sets.

### Western Blot

**Figure 3.**
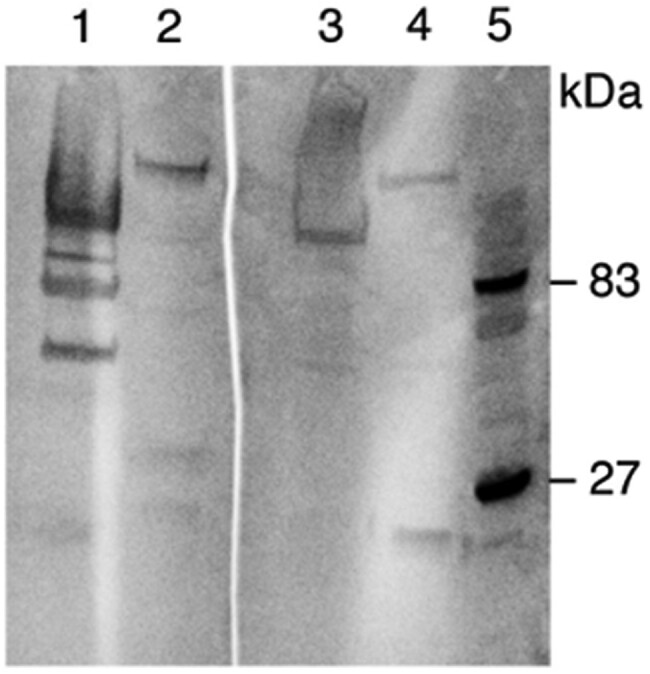
Samples were run on a 4%–12% SDS-polyacrylamide gel, which was electroblotted onto a PVDF membrane. Lanes 1 and 3, *B. divergens* merozoite extract; lanes 2 and 4, extract from O+RBC; lane 5, molecular size marker. The membrane was cut in 2 and incubated with serum from 2 different samples that were positive on ELISA. Abbreviations: ELISA, enzyme-linked immunosorbent assay; RBC, red blood cells; PVDF, polyvinylidene difluoride.

To substantiate the ELISA results, antibody binding to merozoite protein extract was done by Western blotting. The two samples that were strongly positive in our ELISA both recognized protein bands not present in the control RBC extract (Figure 3). The specificities and levels of anti-*Babesia* antibodies in both samples were different as the samples in lane 1 recognized more bands and more strongly; however, both samples recognized a band of ∼120 kDa.

## DISCUSSION

Babesiosis is considered an emerging disease, and the parasite is probably underdiagnosed in humans [6, 33–35]. It is spread by ticks, and with global warming tick-borne diseases can spread further north. In the Northeastern United States, every bag of donated blood is tested for *Babesia* as the parasites can easily spread via blood transfusion, but in Europe there is no testing. It has been shown before that not only *B. microti* but also *B. divergens* can survive for at least 1 month in storage of blood bags in cold temperatures [12].

Maintenance of *B. divergens* growth in cell culture has in this study been used under slightly different circumstances compared with what has been done before. This parasite has in the literature been grown mostly in RPMI 1640 media supplemented with human serum [23, 36, 37], but we used Albumax II, and we could get any desirable parasitemia that we wanted compared with when using human serum. We also found that inclusion of human serum in cultures meant that merozoite antigen preparation could cause the merozoite extract to give unspecific signals in our ELISA, in which case human IgG not completely removed during the washing could be accidentally included in the coating of ELISA plates alongside the merozoite antigens. Also, unlike previous studies that cultured *B. divergens* in a specific gas composition (5% N_2_, 5% O_2_, 90% CO_2_) and sometimes on rotating platforms [38], we used candle light boxes on a stationary platform, which are comparatively cheaper and easier to handle.

Some earlier studies have also used culture supernatant of *B. divergens* as a source of antigens in serological tests, but they focused on secreted/exoantigens [19, 39] or metabolic antigens [20]. We used merozoites that are always present in the supernatants. Some proteins expressed on the surface of *B. microti* merozoites have been found to be highly immunodominant [22]; this is expected due to their contact with the immune system just before they are able to invade new RBC. An earlier study showed that *B. divergens* extracellular merozoites could be viable for up to 1 hour in vitro [36], even though they usually invade new erythrocytes within a few seconds or minutes [40].

The ELISA protocol tested here using crude merozoite protein extract produced satisfactory results, with an almost perfect correlation with IFA dilution factors. While the sensitivity of our method could be lower or comparable with other studies that used different antigens from *B. divergens* or *B. microti*, the specificity is mostly higher or comparable [20, 22, 41]. With a sensitivity of 86% and a specificity of 100%, this ELISA protocol can specifically detect IgG antibodies naturally produced against *B. divergens* and may be used when coinfection is suspected. However, we do not completely rule out the possibility of cross-reactivity when *Plasmodium* or other *Babesia*/piroplasm antigens are present. Acute infections of malaria do not produce strongly positive OD values in our assay, and most of the Ugandan and Nigerian samples are expected to have antibodies against malaria since they are from endemic areas. An earlier ELISA-based protocol similar to the one described here, although using ovine RBC, demonstrated some levels of cross-reactivity with infections due to other *Babesia* and piroplasm species [20]. When using RBC from an animal, there is always a risk of individuals having heterophilic antibodies; this potential source of false positives was avoided in our assay by using human O+RBC for cultures.

We had access to 1 patient sample from an active infection with a *crassa*-like *Babesia*, which is more closely related to *B. divergens* than to *B. microti* [32]. Cross-reactive antibodies between *B. venatorum* and *B. divergens* have been shown before [25], so it is not surprising to see this kind of cross-reactivity also for the *crassa*-like *Babesia*. However, *B. microti* is more distantly positioned in the phylogenetic tree, and antibodies against *B. microti* usually do not cross-react with *B. divergens* antigens [6].

We also included 5 samples from individuals admitted to a Swedish Infectious Disease Clinic due to unknown diagnoses. We included these to investigate whether any activation of the immune system would produce a positive signal in our assay. We found 1 positive sample, which could actually be due to a recent *Babesia* infection, but at least we could see that not all 5 individuals produced a strongly positive signal. In an assay like this, it is difficult to assign the cutoff value for what should be considered a positive or negative sample, as we do not have access to genuinely negative samples; that is, samples from individuals who have never been exposed to any kind of *Babesia*. Also, the possibility that samples may contain antibodies cross-reacting with, for example, *Plasmodium* is complicating the cutoff assessment. In our case, we chose to use “strongly positive” or “weakly positive” to determine who had been exposed, and when using the cutoff for “strongly positive” there was a very good correlation with IFA. “Weakly positive” probably indicates individuals who have been exposed to parasites a while ago and thus are experiencing declining levels of antibodies.

In immunoblotting, we could see that the tested individuals reacted to different antigens, which could be because they have been exposed to different variants of *Babesia*. Another reason could be that one of the individuals was recently exposed, and therefore the antigens were more clearly visible. We believe that the broad spectrum of antigens used in our ELISA is an advantage as more *Babesia* antibodies are likely to be captured compared with when a single or very few recombinant antigens are used.

The goal of our work was to generate a simple and objective method that can be used to detect human antibodies elicited during a natural infection. Through testing different sample sets, we have been able to establish an ELISA protocol that can be used to measure anti-*Babesia* antibodies in humans. However, maintenance of continous cultures is laborious, so it is important to identify the immunodominant antigens in our merozoite crude extract. Further testing of this ELISA protocol is required to establish its usefulness as a tool that can be deployed for research and clinical use, but the present results indicate that it is a very promising alternative to IFA.

## Supplementary Material

ofae028_Supplementary_Data

## Supplementary Data

Supplementary materials are available at *Open Forum Infectious Diseases* online. Consisting of data provided by the authors to benefit the reader, the posted materials are not copyedited and are the sole responsibility of the authors, so questions or comments should be addressed to the corresponding author.
