# Supplementary material for: How to Detect Antibodies Against Babesia divergens in Human Blood Samples
Source: Open Forum Infect Dis. 2024 Jan 16;11(2):ofae028. doi: 10.1093/ofid/ofae028 (PMC10849114; doi:10.1093/ofid/ofae028)
Supplement: ofae028_Supplementary_Data [file ofae028_supplementary_data.zip › Supplementary figure 1 legend.docx]

Supplementary figure 1 legend:

Levels of antibodies against *Babesia divergens* in different groups expressed as OD-level. Horizontal lines indicate limits for considering a sample as strongly or weakly positive.
