# Supplementary figures and images for: How to Detect Antibodies Against Babesia divergens in Human Blood Samples
Source: Open Forum Infect Dis. 2024 Jan 16;11(2):ofae028. doi: 10.1093/ofid/ofae028 (PMC10849114; doi:10.1093/ofid/ofae028)

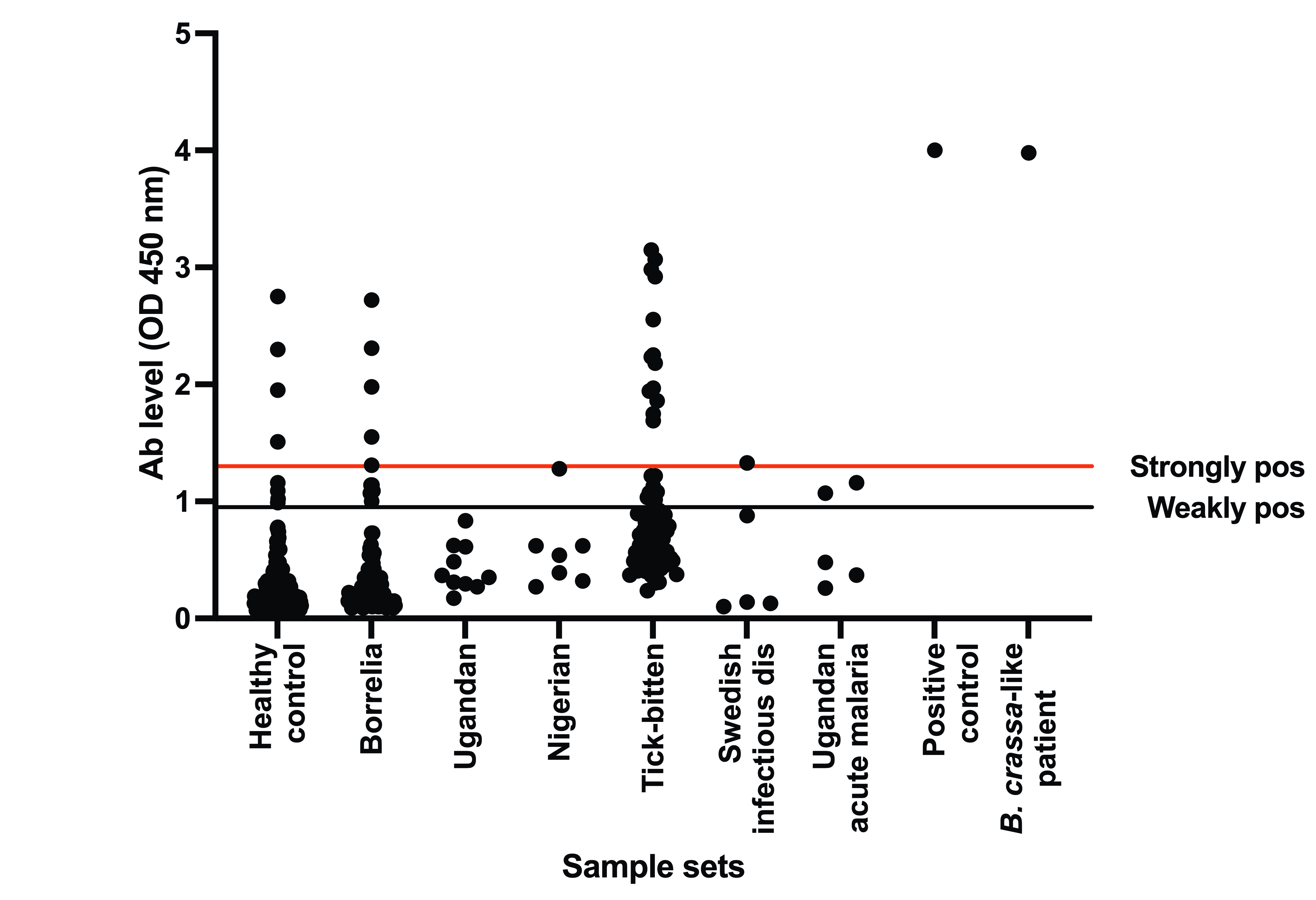

Supplement: ofae028_Supplementary_Data [file ofae028_supplementary_data.zip › 23-12-18-Suppl Figure 1.tiff]
